# The effect of nitrogen availability and water conditions on competition between a facultative CAM plant and an invasive grass

**DOI:** 10.1002/ece3.3296

**Published:** 2017-08-23

**Authors:** Kailiang Yu, Paolo D'Odorico, David E. Carr, Ashden Personius, Scott L. Collins

**Affiliations:** ^1^ Department of Environmental Sciences University of Virginia Charlottesville VA USA; ^2^ Department of Biology University of Utah Salt Lake City UT USA; ^3^ Department of Environmental Science, Policy and Management University of California Berkeley CA USA; ^4^ Department of Biology Villanova University Villanova PA USA; ^5^ Department of Biology University of New Mexico Albuquerque NM USA

**Keywords:** California's coastal grasslands, competition, crassulacean acid metabolism, invasive grass, *Mesembryanthemum crystallinum*, nutrient, water stress

## Abstract

Abstract Plants with crassulacean acid metabolism (CAM) are increasing their abundance in drylands worldwide. The drivers and mechanisms underlying the increased dominance of CAM plants and CAM expression (i.e., nocturnal carboxylation) in facultative CAM plants, however, remain poorly understood. We investigated how nutrient and water availability affected competition between *Mesembryanthemum crystallinum* (a model facultative CAM species) and the invasive C_3_ grass *Bromus mollis* that co‐occur in California's coastal grasslands. Specifically we investigated the extent to which water stress, nutrients, and competition affect nocturnal carboxylation in *M. crystallinum*. High nutrient and low water conditions favored *M. crystallinum* over *B. mollis*, in contrast to high water conditions. While low water conditions induced nocturnal carboxylation in 9‐week‐old individuals of *M. crystallinum*, in these low water treatments, a 66% reduction in nutrient applied over the entire experiment did not further enhance nocturnal carboxylation. In high water conditions *M. crystallinum* both alone and in association with *B. mollis* did not perform nocturnal carboxylation, regardless of the nutrient levels. Thus, nocturnal carboxylation in *M. crystallinum* was restricted by strong competition with *B. mollis* in high water conditions. This study provides empirical evidence of the competitive advantage of facultative CAM plants over grasses in drought conditions and of the restricted ability of *M. crystallinum* to use their photosynthetic plasticity (i.e., ability to switch to CAM behavior) to compete with grasses in well‐watered conditions. We suggest that a high drought tolerance could explain the increased dominance of facultative CAM plants in a future environment with increased drought and nitrogen deposition, while the potential of facultative CAM plants such as *M. crystallinum* to expand to wet environments is expected to be limited.

## 1 INTRODUCTION

Climate change studies predict an intensification of drought in many drylands around the world (Easterling et al., 2000; IPCC 2013). Human activities associated with fertilizer applications have dramatically increased atmospheric nitrogen deposition, a trend that is expected to continue in the decades to come (Goulding et al., 1998; Galloway et al., 2008). These key global change drivers (i.e., altered water and nutrient conditions) have been found to greatly affect ecological processes such as interspecific interactions in ecosystems where C_3_ and/or C_4_ plants dominate (Niu, Liu, & Wan, 2008; Van der Waal et al., 2009). It remains unclear, however, how water and nutrient conditions affect plants with crassulacean acid metabolism (CAM) and their competitive relationship with other functional types (but see Yu & D'Odorico, 2015, 2017; Yu, D'Odorico, Li, & He, 2017).

Crassulacean acid metabolism, a unique photosynthetic pathway evolving from C_3_ photosynthesis, is expressed by ~6%–7% of vascular plant species (Smith & Winter, 1996; Crayn, Smith, & Winter, 2004). CAM plants feature nocturnal CO_2_ uptake, water storage, and a high water use efficiency (Lüttge, 2004; Borland, Barrera Zambrano, Ceusters, & Shorrock, 2011). Obligate CAM species perform nocturnal carboxylation independently of environmental conditions, while the behavior of facultative CAM plants depends on environmental drivers (i.e., water stress; Lüttge, 2004; Borland et al., 2011). Because of its photosynthetic plasticity and water‐conserving mode, crassulacean acid metabolism has been recognized as one of the most intriguing plant adaptations to water stress (Cushman & Borland, 2002; Winter & Holtum, 2007), which provides CAM plants with ecological opportunities to increase their abundance in a changing environment (Drennan & Nobel, 2000; Cushman & Borland, 2002; Borland, Griffiths, Hartwell, & Smith, 2009; Reyes‐García, 2009).

This study investigated the effects of nutrient and water conditions on competition between the model facultative CAM species *Mesembryanthemum crystallinum* and its C_3_ competitor *Bromus mollis,* which co‐occur in California's coastal grasslands (Vernon, Ostrem, Schmitt, & Bohnert, 1988; Schmitt, 1990; Winter & Holtum, 2014). *M. crystallinum* is native to southern and eastern Africa and was introduced to, and then spread throughout, western Australia, the Mediterranean basin, and along the coasts of the western United States, Mexico, and the Caribbean (Adams et al., 1998). In natural habitats, it germinates and establishes in the rainy season with C_3_ photosynthesis and then switches to CAM photosynthesis in response to environmental stress (e.g., low water and/or high salinity). This switch in photosynthetic pathway occurs when *M. crystallinum* transitions from juvenile to adult (≈>6–7 weeks old) and develops secondary/succulent leaves alongside shoots (Osmond, 1978; Winter & Holtum, 2007, 2014). This CAM behavior in *M. crystallinum* can revert back to C_3_ photosynthesis after removing the source of environmental stress, thus demonstrating the crucial role of environmental controls in CAM behavior (Vernon et al., 1988; Schmitt, 1990; Winter & Holtum, 2014). Field observations show that *M. crystallinum* appears to be increasing its abundance in coastal California (Vivrette & Muller, 1977; Corbin & D'Antonio, 2009), while its potential expansion in a future changing environment (i.e., increased drought and nitrogen deposition) remains unclear.

*Mesembryanthemum crystallinum* with high drought tolerance would be expected to outcompete *B. mollis* in a future drier climate. Increased N deposition would increase high growth rates and water usage of grasses (*B. mollis*) in the wet (rainy) season (McCown & Williams, 1968), which increases water stress of grasses in the subsequent dry season, and thus potentially favors *M. crystallinum* by releasing competitive pressure from grasses. In comparison, in consistently wet conditions *B. mollis* could sustain higher growth rates, especially in high N conditions, and thus have a competitive advantage with respect to *M. crystallinum* in access to light, soil water, and nutrients. This competitive advantage by *B. mollis* may exert biotic stress on *M. crystallinum*. However, it remains unclear whether *M. crystallinum* may adapt to biotic stress from *B. mollis* by switching to CAM photosynthesis, a strategy of photosynthetic plasticity found to increase its reproduction rate and fitness (Winter & Ziegler 1992; Cushman et al., 2008; Herrera, 2009). Testing these novel hypotheses would provide new insights into crassulacean acid metabolism as an adaptive strategy to both abiotic and biotic stress. The ability of facultative CAM plants to adapt to strong competition enhances their potential to expand in wet environments, an aspect that has been largely ignored in the past decade.

Another knowledge gap is the effects of nutrient availability (mainly N) on CAM expression in *M. crystallinum*. Some studies in obligate CAM species indicate that N deficiency limited the rate of CAM photosynthesis (Winter, Foster, Schmitt, & Edwards, 1982; Nobel, 1983), presumably because of the N requirements by the enzymes used for photosynthesis. In contrast, other studies show that CAM plants (including both obligate and facultative) grown in conditions with lower N availability had a higher CAM expression (Ota, 1988; Paul & Cockburn, 1990; Lüttge, 2006; Winter & Holtum, 2011). Recent studies recognized the role of carbohydrates (i.e., 3‐carbon acceptor phosphoenolpyruvate, PEP which is produced by degrading starch/sugars) as substrates in nocturnal carboxylation (Borland & Dodd, 2002; Antony & Borland, 2008; Antony et al., 2008). Haider, Barnes, Cushman, and Borland (2012) found that CAM expression in a starch‐deficient mutant of *M. crystallinum* was suppressed under high salt additions. Indeed, the only study to investigate the effects of N and P deficiency on CAM expression in *M. crystallinum* found that CAM expression increased (Paul & Cockburn, 1990). However, the N and P deficiency was applied at the adult stage, after the plants were able to accumulate a sufficient amount of carbohydrates from earlier growth stages with no N and P limitation. It is unclear how nutrient treatments applied in early life stages and their interactions with water availability could affect CAM expression in *M. crystallinum*.

We conducted greenhouse experiments in which the seedlings of *M. crystallinum* and *B. mollis* in both monoculture and mixtures were subjected to two nutrient levels (“high” and “low”) and two water levels (“high” and “low”). Plant responses were evaluated through measurements of gas exchange, concentrations of titratable acidity, aboveground plant N, biomass, and productivity. We asked: (i) How does competition between *M. crystallinum* and *B. mollis* respond to nutrient and water conditions? (ii) Is the physiological plasticity of facultative CAM in *M. crystallinum* an adaptive strategy for competition with *B. mollis*? (iii) How does nutrient availability and its interactions with water availability influence CAM expression and reversibility in *M. crystallinum*?

## 2 MATERIALS AND METHODS

### 2.1 Experimental design

*Mesembryanthemum crystallinum* seeds were germinated in plastic trays covered with 1–2 mm substratum of mineral soil in the greenhouse facility at the University of Virginia. Likewise, seeds of *B. mollis* were germinated in plastic pots (14.5 cm in diameter and 10.5 cm in height with a capacity of 1.3 L) in the greenhouse. Germination started on 6 September 2015, and by 20 September 2015, seedlings of *M. crystallinum* and *B. mollis* were ready to be transplanted in either monoculture (one individual of *M. crystallinum* or 20 individuals of *B. mollis*) or a mixture (one individual of *M. crystallinum* and 20 individuals of *B. mollis* with *M. crystallinum* located in the middle of each pot). A mixture of Canadian sphagnum peat moss and calcined clay (3:2) was used; this soil had high hydraulic conductivity to simulate sandy conditions in California's coastal grasslands.

**Figure 1:**
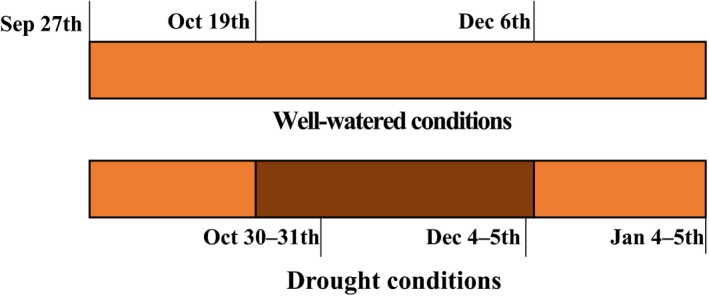
Schematic diagram of water treatments in high and low water conditions. Black zone represents low‐frequency watering treatment (once every 8 days) while the white zone represents high‐frequency watering treatment (once every 2 days). Plants were harvested in the first (October 30–31th), second (December 4–5th), and third (January 4–5th) stages of the experiment. Gas exchange and titratable acidity were measured 1–2 days before each harvest

The study used a randomized block experiment design in which the seedlings of *M. crystallinum* and *B. mollis* in both monoculture and mixture were subjected to two nutrient fertilization levels (high and low) under high and low water conditions. This experiment had three stages of plant harvest (Figure 1), and there were six replicates arranged in six blocks for each measurement in each stage. Fertilizer was applied in the form of Peters Professional 20‐20‐20 (20% total N including 3.2% NH_4_‐N, 5.3% NO_3_‐N, and 11.5% urea, 20% P_2_O_5_, 20% K_2_O, as well as other micronutrients). Each pot in high nutrient conditions received 15 mg N once every 8 days while each pot in low nutrient conditions received 15 mg N once every 24 days. Plants in high water conditions were watered every 2 days with an intensity of 8 mm per event during the whole experiment. Plants in low water conditions were watered every 2 days with an intensity of 8 mm per event until 19 October 2015 and were then watered every 8 days with an intensity of 8 mm per event until 6 December 2015, when the water treatment returned to high water conditions (i.e., watering frequency of once every 2 days) until the end of the experiments (5 January 2015); thus, this low water treatment was in the form of a “wet–dry–wet” sequence (Figure 1). We note that, in contrast to previous studies (Winter & Holtum, 2007, 2014), the low water treatment entailed a low watering frequency instead of complete interruption of water applications. The competitive advantage of *M. crystallinum* over *B. mollis* and its successful invasion of coastal California has been attributed to its high salt tolerance (Vivrette & Muller, 1977). Over this experiment salts were not added to increase soil salinity because the purpose of this study was to investigate the potential expansion of *M. crystallinum* under increased drought and nitrogen deposition. Plants were subjected to well controlled light (i.e., 12‐hr light with photosynthetically photon flux density (PPFD) of 700–800 μmol m^−2^ s^−1^ from 7 a.m. to 7 p.m. EDT) and temperature (i.e., 25°C during the day and 20°C during the night) conditions.

### 2.2 Gas exchange and titratable acidity measurement

Over the experiment, *M. crystallinum* produces regular/secondary leaves, which allows the measurements of gas exchange using a standard leaf chamber (2 × 3 cm^2^) in a Licor 6400 gas analyzer. Before plant harvest (usually 2 days before), gas exchange for leaves of *M. crystallinum* and *B. mollis* in both monoculture and mixture were measured using the standard Licor leaf chamber. During the day, the measurements of gas exchange were made for *M. crystallinum* and *B. mollis* between 10:00 a.m. and 12:00 p.m. at a constant leaf temperature of 23°C and photosynthetically active radiation of 1,500 μmol m^−2^ s^−1^; during the night, gas exchange was measured once every 2 hr between 8 p.m. and 8 a.m. on the following day for *M. crystallinum*—both alone and mixed with *B. mollis*—at a constant leaf temperature of 23°C and photosynthetic active radiation of 0 μmol m^−2^ s^−1^. Thus, gas exchange was measured once during the day and several times at night to capture the nocturnal change of CO_2_ uptake.

After measurements of gas exchange (still before plant harvest), in each treatment six leaves from six individuals of *M. crystallinum* (alone or mixed with *B. mollis*; one leaf each individual) were sampled from each plant at 7 a.m. and 5 p.m., respectively, and then stored at −20°C before measurements of titratable acidity. CAM photosynthesis is characterized by a temporal separation of the dark (i.e., accumulation of 4‐C organic acids using phosphoenolpyruvate carboxylase to fix CO_2_) and light reactions of photosynthesis (i.e., decarboxylation of 4‐C organic acids for Calvin cycle using Rubisco). Thus, a significant increase in titratable acidity overnight indicates the occurrence of CAM photosynthesis in *M. crystallinum*. Titratable acidity was measured using the acid base titration method (Von Caemmerer & Griffiths, 2009), whereby leaf disks (4 cm^2^) are boiled in 1.5 ml H_2_O for 5 min in a microfuge tube; 10 mmol/L NaOH was added into the same tube with 20 μl of a 1/5 dilution of phenolphthalein as indicator. Titratable acidity was then calculated from the amount of NaOH added (Von Caemmerer & Griffiths, 2009).

### 2.3 Light availability and biomass measurements

Before plant harvest, light intensity in mixture was measured above and at the bottom of canopies (approximately at ground level) using a HOBO Pendant® Temperature/Light 64K Data Logger. Relative light intensity (%) was calculated as the ratio of light intensity under canopies to that above canopies (Sun et al. 2016). Plants were harvested on October 30–31th (the first stage), December 4–5th (the second stage), and January 4–5th (the third stage), respectively, with six replicates in each block in each stage (Figure 1). *Mesembryanthemum crystallinum* and *B. mollis* in mixture were separated; loose roots found in the soil profile not attached to the parent plant (<5% of total root biomass) were classified as belonging to *M. crystallinum* or *B. mollis* based on root color, diameter, and shape. Roots were washed free of soil through 0.1‐mm mesh sieves. Plant tissues were dried at 60°C for 72 hr and weighted. Total biomass and shoot‐to‐root biomass ratios were calculated. Note that the samples of fresh *M. crystallinum* collected for measurements of titratable acidity were weighted and then converted to dry biomass using the fresh/dry biomass ratio, based on our measurements.

### 2.4 Plant leaf water potential and plant N content

Plant leaf water potential was measured using a Decagon WP4® potentiometer. Plant samples dried at 60°C for 72 hr were ground and homogenized for elemental analysis. Plant N analysis was performed using a Thermo Scientific FLASH 2000 NC Analyzer.

### 2.5 Statistical analysis

The effects of nutrient treatment, water availability, species competition and time, as well as their interactions, on plant leaf water potential (LWP), specific leaf area (SLA), diurnal photosynthetic assimilation (A_D_), total biomass (TB), belowground‐to‐aboveground biomass ratio (BA), and aboveground plant total N (APN) were analyzed using a five‐way ANOVA with block as a random factor. The effects of nutrient, water, species, and time as well as their interactions on soil moisture were analyzed using a four‐way ANOVA with block as a random factor. The BA values were natural log transformed prior to ANOVA. In general, the most interesting effects were found in multiway interactions. To explore these interactions, we constructed pairwise orthogonal contrasts to detect differences between individual pairs of means. All statistics were performed in SAS 9.4.

## 3 RESULTS

### 3.1 Competition between *Mesembryanthemum crystallinum* and *Bromus mollis*

**Figure 2:**
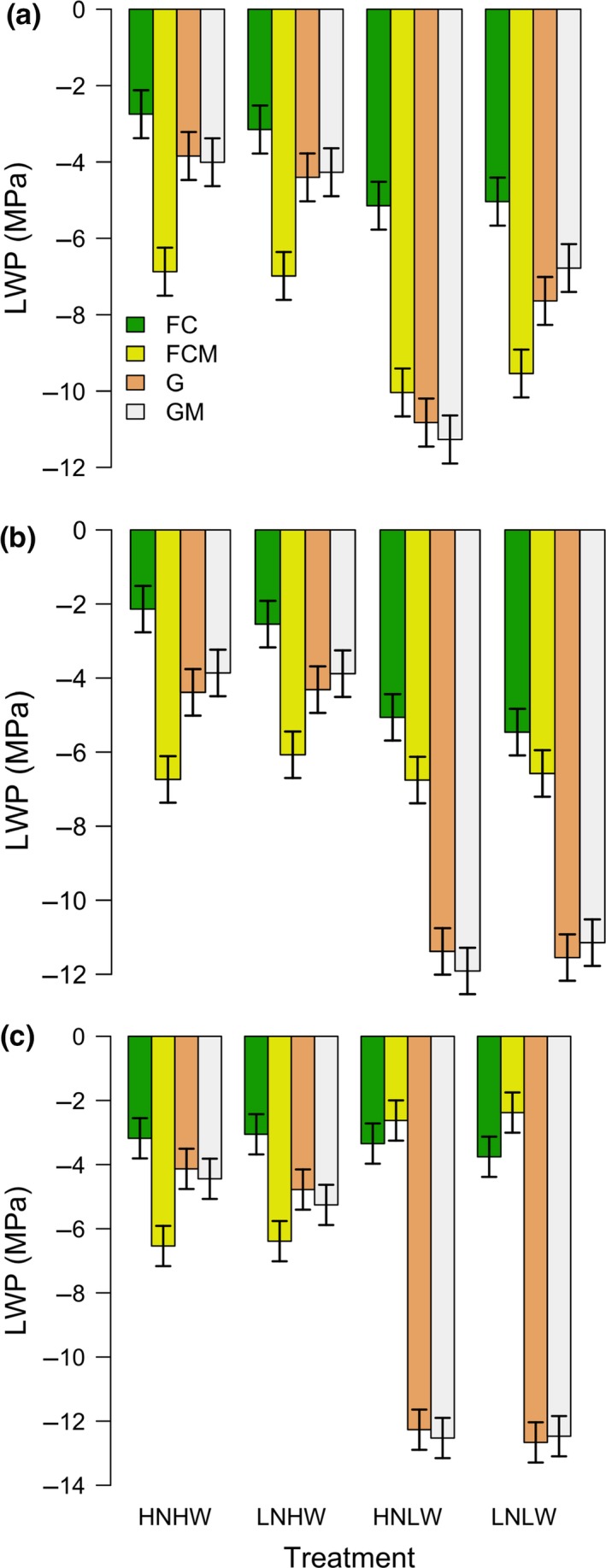
Leaf water potential (LWP) in *Mesembryanthemum crystallinum* alone (FC), *M. crystallinum* in mixture (FCM), *Bromus mollis* alone (G), *B. mollis* in mixture (GM) under different nutrient and water conditions in the first (a), second (b), and third (c) stages of the experiment. HNHW, high nutrient and high water conditions; LNHW, low nutrient and high water conditions; HNLW, high nutrient and low water conditions; LNLW, low nutrient and low water conditions. Each bar represents the mean of six values while error bars indicate 95% confidence intervals

**Figure 3:**
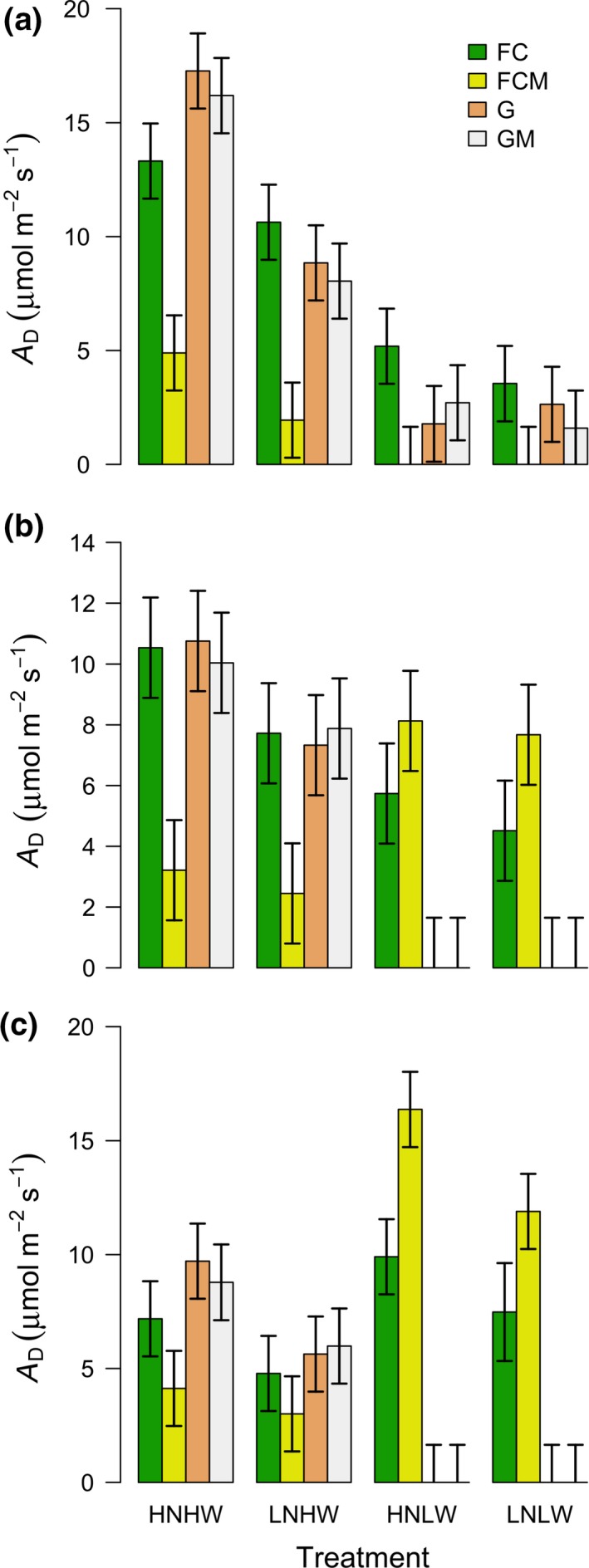
Photosynthetic assimilation during the day (A_D_) in *Mesembryanthemum crystallinum* alone (FC), *M. crystallinum* in mixture (FCM), *Bromus mollis* alone (G), *B. mollis* in mixture with *M. crystallinum* (GM) under different nutrient and water conditions in the first (a), second (b), and third (c) stages of the experiment. Symbols for each treatment are the same as Figure 2. Each bar represents the mean of six values while error bars indicate 95% confidence intervals

**Figure 4:**
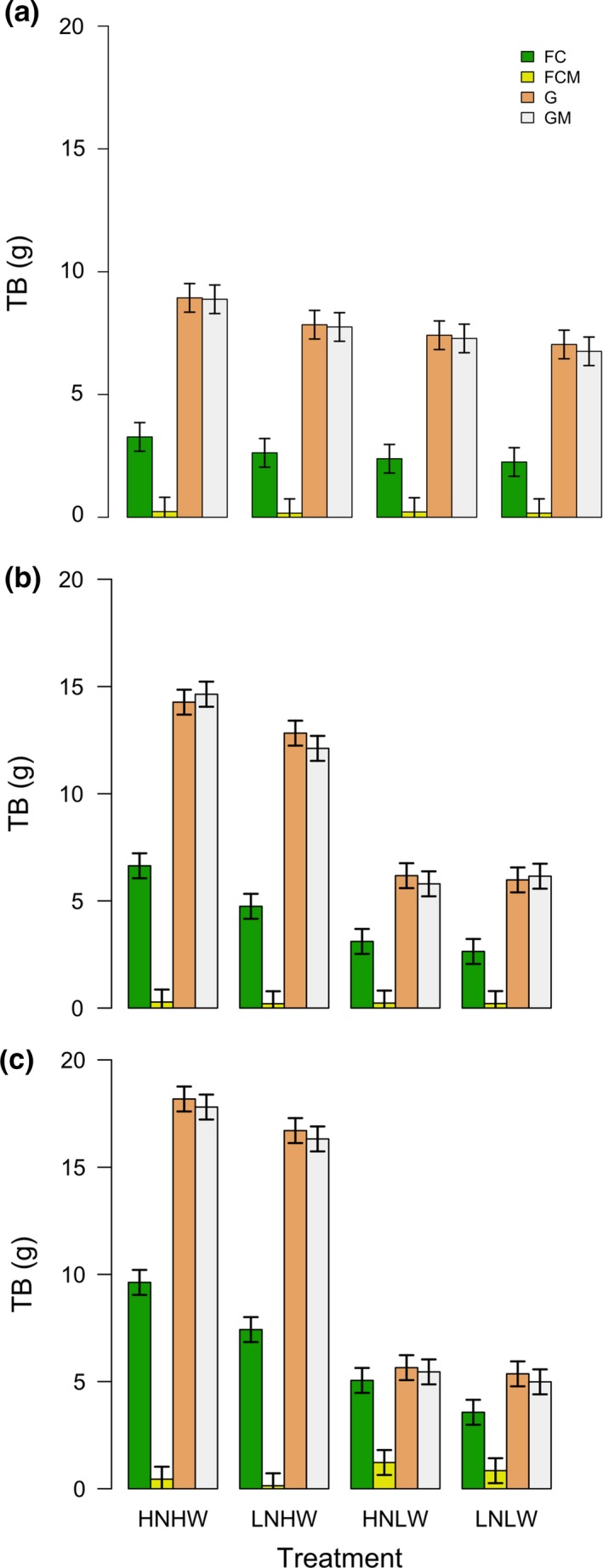
Total biomass (TB) in *Mesembryanthemum crystallinum* alone (FC), *M. crystallinum* in mixture (FCM), *Bromus mollis* alone (G), *B. mollis* in mixture (GM) under different nutrient and water conditions in the first (a), second (b), and third (c) stages of the experiment. Symbols for each treatment are the same as Figure 2. Each bar represents the mean of six values while error bars indicate 95% confidence intervals

Leaf water potential in *M. crystallinum* alone (FC) was significantly greater than in *B. mollis* alone (G) and *B. mollis* in mixture (GM), especially in low water conditions over all stages of the experiment (all *p* ≤ 0.0158, Figure 2). *B. mollis* both alone and in mixture died at some time between the first and second stage, as shown by its extremely low leaf water potential (Figure 2a,b), lack of photosynthetic assimilation (Figure 3a,b), and lack of increase in total biomass (Figure 4b,c) in the second and third stages of the experiment. Overall, these results indicate that low water conditions favor *M. crystallinum* over *B. mollis*. The competitive advantage of *M. crystallinum* over *B. mollis* in low water conditions was improved after nitrogen addition, as evidenced by a sharp decrease in leaf water potential of *B. mollis* both alone and in mixture in high nutrient, low water conditions (HNLW; LWP = −0.8/−11.3 MPa for G/GM) versus low nutrient, low water conditions (LNLW; LWP = −7.6/−6.8 MPa for G/GM; *p* < 0.0001, Figure 2a). In fact, *B. mollis* both alone and in mixture in HNLW died earlier than LNLW, thus releasing its competitive pressure on *M. crystallinum* in mixture. In high water conditions, *B. mollis* both alone and in mixture sustained high photosynthetic assimilation (Figure 3a,b) and biomass (Figure 4a,b). This exerted strong competitive effects of *B. mollis* on *M. crystallinum* in mixture in access to soil nutrients and light (Figs S1 and S2), thus leading to consistently lower leaf water potential (Figure 2a,b), photosynthetic assimilation (Figure 3a,b), and biomass (Figure 4a,b) in *M. crystallinum* mixed with *B. mollis* than *M. crystallinum* alone over the three stages of the experiment. Overall, these results show the competitive advantage of *B. mollis* over *M. crystallinum* in high water conditions.

### 3.2 Effects of nutrient availability, water conditions, and competition on plant response

Leaf water potential (Figure 2), photosynthetic assimilation (Figure 3), and total biomass (Figure 4) of all vegetation types were generally greater in high water conditions than low water conditions regardless of nitrogen conditions in the first and second stages of the experiment. The exceptions, however, are the cases of *M. crystallinum* in mixture where competition outweighed the water effects (i.e., in terms of leaf water potential, both *p* ≥ 0.2736 in the second stage, Figure 2b; in terms of total biomass, all *p* ≥ 0.8514 in the first and second stages). Similarly, competition outweighed the nitrogen effects in some cases, as evidenced by lack of significant increase in photosynthetic assimilation and total biomass in high nitrogen conditions as compared to low nitrogen conditions in the second and third stages of the experiment (Figures 3 and 4). This competitive effect (when grasses were alive) also led to a lower leaf water potential (Figure 2), photosynthetic assimilation (Figure 3), and total biomass (Figure 4) in *M. crystallinum* in mixture than *M. crystallinum* alone.

There was a significant effect of nutrient and water interactions in affecting leaf water potential, photosynthetic assimilation, and total biomass (Table 1; *p* < 0.001 for N × water). Similar to the pattern of specific leaf area (Fig. S3), in high water conditions, A_D_ of all vegetation types in high nutrient conditions was significantly greater than low nutrient conditions (all *p* ≤ 0.0387, Figure 3). In low water conditions, A_D_ of all vegetation types in high nutrient conditions was not significantly different from low nutrient conditions in the first and second stages (all *p* ≥ 0.1675, Figure 3a,c). For high water conditions, an increase in nutrient availability significantly increased total biomass of *M. crystallinum* alone (FC), *B. mollis* alone and *B. mollis* in mixture in all three stages of the experiment (all *p* ≤ 0.0006, Figure 4) except the case of FC in the first stage (*p* = 0.122, Figure 4a). In low water conditions, an increase in nutrient availability significantly increased total biomass of *M. crystallinum* alone in the third stage (a high water treatment; *p* = 0.0004, Figure 4c), in contrast to other cases (all *p* ≥ 0.2667, Figure 4).

**Table 1:** Results (*p* values) of five‐way factorial ANOVA on total biomass (TB), belowground‐to‐aboveground biomass ratio (BA), photosynthetic assimilation during the day (A_D_), plant leaf water potential (LWP), specific leaf area (SLA), and aboveground plant N (APN)

	*df*	TB	BA	A_D_	LWP	SLA	APN
Nutrient	1	<0.0001	<0.0001	<0.0001	0.0075	<0.0001	<0.0001
Water	1	<0.0001	<0.0001	<0.0001	<0.0001	<0.0001	0.0001
Species	1	<0.0001	<0.0001	<0.0001	<0.0001	–	0.0004
Competition	1	<0.0001	<0.0001	<0.0001	<0.0001	<0.0001	<0.0001
Time	2	<0.0001	<0.0001	0.0249	–	<0.0001	<0.0001
Nutrient × water	1	<0.0001	<0.0001	<0.0001	<0.0001	<0.0001	0.0023
Nutrient × species	1	–	0.0227	–	0.0139	–	0.0002
Nutrient × competition	1	0.0192	–	–	0.0098	–	–
Nutrient × time	2	–	–	0.0211	<0.0001	–	<0.0001
Water × species	1	<0.0001	<0.0001	<0.0001	<0.0001	<0.0001	<0.0001
Water × competition	1	<0.0001	<0.0001	<0.0001	<0.0001	0.0002	0.0017
Water × time	2	<0.0001	<0.0001	<0.0001	<0.0001	<0.0001	<0.0001
Species × competition	1	<0.0001	<0.0001	<0.0001	<0.0001	<0.0001	<0.0001
Species × time	2	<0.0001	–	<0.0001	<0.0001	<0.0001	<0.0001
Competition × time	2	<0.0001	<0.0001	<0.0001	<0.0001	0.0008	0.0089
Nutrient × water × species	1	0.0115	0.0213	<0.0001	<0.0001	–	0.0009
Nutrient × water × competition	1	–	0.0038	–	–	–	–
Nutrient × water × time	2	–	–	0.0012	<0.0001	–	0.033
Nutrient × specie × competition	1	0.0007	0.0035	–	–	–	–
Nutrient × species × time	2	–	–	0.0188	<0.0001	0.0046	0.0005
Nutrient × competition × time	2	–	–	–	–	–	–
Water × specie × competition	1	<0.0001	<0.0001	<0.0001	<0.0001	<0.0001	0.0402
Water × species × time	2	<0.0001	<0.0001	<0.0001	<0.0001	<0.0001	<0.0001
Water × competition × time	2	<0.0001	<0.0001	–	<0.0001	0.0099	0.0341
Species × competition × time	2	<0.0001	<0.0001	<0.0001	<0.0001	<0.0001	0.0009
Nutrient × water × species × competition	1	0.0408	–	–	–	–	–
Nutrient × water × species × time	2	–	–	–	0.0006	–	–
Nutrient × water × competition × time	2	–	–	–	–	–	–
Nutrient × species × competition × time	2	–	–	–	–	–	0.0224
Water × species × competition × time	2	<0.0001	0.0238	0.0456	<0.0001	0.0112	–
Nutrient × water × species × competition × time	2	–	–	–	–	–	–

“–” means not significant (*p* > 0.05).

### 3.3 Nocturnal photosynthetic assimilation and titratable acidity of CAM plants

**Figure 5:**
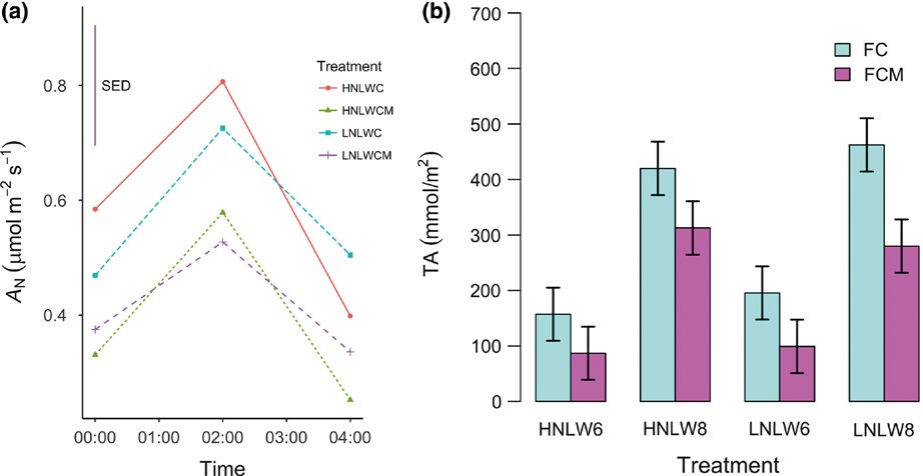
(a) Nocturnal change of photosynthetic assimilation (A_N_) of *Mesembryanthemum crystallinum* in alone (FC) and mixture (FCM) in low water conditions in the second stage of the experiment. Symbols for each treatment are the same as in Figure 2. (b) Titratable acidity (TA) of *M. crystallinum* alone (FC) and in mixture (FCM) in low water conditions in the second stage. Six refers to 6 p.m. while eight refers 8 a.m. “SED”, the standard deviation of samples. Both FC and FCM do not express CAM behavior in any treatments during the first and third stages of the experiment, and thus values of A_N_ and TA in these two stages are not shown

*Mesembryanthemum crystallinum* (either alone or mixed with *B. mollis* grasses) did not perform CAM expression (nocturnal carboxylation) in the first stage of any of the treatments, as indicated by the negative values of nocturnal photosynthetic assimilation (A_N_ ≈ −1–2 μmol m^−2^ s^−1^) and the lack of nocturnal accumulation of titratable acidity. In the second stage, the A_N_ of *M. crystallinum* (both alone and in mixture) was also negative (≈−1–2 μmol m^−2^ s^−1^) in high water conditions, which was consistent with the fact there was no significant difference of titratable acidity (TA) between late afternoon (TA = 48.08/53.98 mmol m^−2^) and early morning (TA = 47.61/51.37 mmol m^−2^). These results indicate that *M. crystallinum* in mixture did not switch to CAM photosynthesis in response to strong competition with *B. mollis*. In contrast, low water treatments in both high nutrient and low nutrient conditions led to a positive A_N_ (A_N_ ≈ 0.5–0.8 μmol m^−2^ s^−1^ by *M. crystallinum* alone and A_N_ ≈ 0.3–0.6 μmol m^−2^ s^−1^ by *M. crystallinum* in mixture) at 1–4 a.m. (Figure 5a) as well as greater TA in early morning than late afternoon (all *p* < 0.0001, Figure 5b), which indicated CAM expression. Reduction of nutrient application in *M. crystallinum* (alone or in mixture) did not significantly affect its A_N_ and titratable acidity in late afternoon and early morning in low water treatments (all *p* ≥ 0.2548, Figure 5). Consistent with the pattern of A_N_, titratable acidity in *M. crystallinum* in mixture was significantly lower than in *M. crystallinum* alone in both late afternoon and early morning in both HNLW and LNLW (both *p* ≤ 0.0411, Figure 5). *M. crystallinum* (either alone or mixed with *B. mollis* grasses) did not perform CAM expression in the third stage (see legend in Figure 5), which indicates that CAM photosynthesis was reverted back to C_3_ photosynthesis after removing environmental stress.

## 4 DISCUSSION

### 4.1 Competition between *Mesembryanthemum crystallinum* and *Bromus mollis*

We found that *M. crystallinum* outcompeted *B. mollis* in low water treatments regardless of nutrient availability (Figures 2, 3, 4). The death of *B. mollis* in low water treatments was mainly caused by its intolerance to water stress exacerbated by the relatively high density (20 individuals per pot) and high biomass accumulated during prior high water conditions, which led to high evapotranspiration. As compared to *B. mollis*,* M. crystallinum* had much higher leaf water potential (Figure 2) and was more tolerant to water stress likely because of its ability to store the absorbed water in aboveground biomass (Lüttge, 2004; Borland et al., 2009). While previous investigations have shown how the higher salt tolerance of *M. crystallinum* could account for its competitive advantage and ability to invade coastal grasslands (Vivrette & Muller, 1977), our study shows how low water conditions could improve the competitive advantage of *M. crystallinum* with respect to *B. mollis*, which *M. crystallinum* coexists with in California. Because droughts are predicted to become more intense across this region (Easterling et al., 2000; IPCC 2013), dominance by CAM plants, such as *M. crystallinum,* will likely increase.

In addition to drought intensification, another driver of environmental change is increased N deposition. Previous studies suggested that Mediterranean ecosystems such as California grasslands where *M. crystallinum* and *B. mollis* interact could be particularly vulnerable to impacts from climate change and N deposition (Parton, Ojima, & Schimel, 1994; Sala, 2000). This study has shown that increased nutrient availability and low water availability affected the competitive relationship between *M. crystallinum* and *B. mollis*. In fact, we found that after ceasing water applications (stage 1 of the experiment) the leaf water potential of *B. mollis*—both alone and in mixture—was much lower with high rates of nutrient supply than in low nutrient conditions (Figure 3a); we also observed that *B. mollis* in high nutrient conditions died earlier (≈1–2 weeks) than in low nutrient conditions in response to drought treatments. Relatively high levels of nutrient availability increased the biomass of *B. mollis,* and consequently led to higher evapotranspiration rates and associated soil moisture depletion, thereby enhancing plant water stress after watering frequency was reduced, consistent with other studies (Zavaleta et al., 2003; Harpole, Potts, & Suding, 2007).

Nutrient level and water co‐limited the photosynthesis and productivity of *M. crystallinum* and *B. mollis* (Figures 3 and 4). These results were consistent with various studies of grasslands across a large range of precipitation regimes (Harpole et al., 2007; Eskelinen & Susan 2015). Moreover, we found much lower leaf water potential (Figure 2), aboveground plant N (Fig. S1), light availability (Fig. S2), photosynthetic assimilation, and total biomass (Figures 3 and 4) in *M. crystallinum* in mixture than alone; thus, *B. mollis* exerted a strong competition effect on *M. crystallinum* for access to soil nutrients and light in high water conditions. This competition effect even outweighed the positive direct effects of increased nutrient availability on photosynthetic assimilation and total biomass of *M. crystallinum* (Figures 3 and 4) and the reduction in root/shoot ratio (Fig. S4). In fact, high‐stature *B. mollis* took advantage of increased nutrient availability and constrained the growth of low‐stature *M. crystallinum* by enhancement of shade effects (Yang et al., 2011; Sun, Yu, Shugart, & Wang, 2015). In agreement with other studies (Tilman, 1988; Lane, Coffin, & Lauenroth, 2000; Harpole et al., 2007), *M. crystallinum* in response to light competition increased the biomass allocation to aboveground (Fig. S4), suggesting a shift in limiting resources from belowground (nutrients) to aboveground (light).

### 4.2 CAM expression and reversibility in *Mesembryanthemum crystallinum* as affected by competition

Surprisingly, in high water conditions, *M. crystallinum* in mixture did not switch from C_3_ photosynthesis to CAM expression over the entire experiment, a type of physiological plasticity *M. crystallinum* typically uses to adapt to environmental stress (Osmond, 1978; Winter & Holtum, 2007, 2014) and increase production of seeds and overall fitness (Cushman et al., 2008; Herrera, 2009). As discussed above, it is possible that light competition outweighed the effect of water competition on *M. crystallinum* in mixture with *B. mollis*. High‐frequency watering (once every 2 days) in high water conditions may alleviate the water stress of *M. crystallinum* even if it is competing with *B. mollis*. Other studies, however, indicated that even moderate water stress can induce CAM expression in *M. crystallinum* and that this effect increases with plant age (Winter & Holtum, 2007, 2014).

Alternatively, in high water conditions photosynthesis and productivity of *M. crystallinum* in association with *B. mollis* was substantially suppressed (Figures 3 and 4) and thus did not have sufficient carbohydrates reserves (Antony & Borland, 2008; Antony et al., 2008) to switch to CAM expression in response to water stress. Similarly, CAM expression under salt stress was found to be suppressed in a starch‐deficient mutant of *M. crystallinum* (Haider et al., 2012) likely because, to maintain metabolism and growth, plants need to partition carbohydrates into other sinks, which compete with the substrate requirement by nocturnal carboxylation (Borland & Dodd, 2002). Moreover, it was also observed that in high water conditions, *M. crystallinum* (mixed with *B. mollis*) did not develop secondary leaves, a trait indicating the transition to adult stage in which CAM expression may be induced (Adams et al., 1998; Winter & Holtum, 2007). This is in contrast to the case of low water treatments (Figure 5), in which *M. crystallinum* reached the adult stage and was therefore capable of developing CAM expression. These results may stress the importance of plant maturity in terms of sufficient carbohydrates instead of plant age in affecting CAM expression in *M. crystallinum*. These results also indicate that the ability of *M. crystallinum* to adapt to strong competition by switching to CAM photosynthesis could be restricted in high water conditions.

### 4.3 CAM expression and reversibility in *Mesembryanthemum crystallinum* as affected by abiotic factors

Our research shows that CAM expression in *M. crystallinum* was the result of environmental controls in response to droughts, consistent with other studies (Vernon et al., 1988; Schmitt, 1990; Piepenbrock & Schmitt, 1991; Winter & Holtum, 2007, 2014). This was demonstrated both by the lack of CAM expression in *M. crystallinum* during the whole three stages in high water conditions, and the reversibility of CAM expression after returning to high water conditions in low water treatments.

Previous studies investigated the effects of N deficiency and its interactions with light on CAM expression in CAM plants, but they reported a mixed response: in some cases, N deficiencies had a negative and in others a positive effect on CAM photosynthesis (Winter et al., 1982; Nobel, 1983; Ota, 1988). Our study shows that in low water conditions, there was no significant difference in CAM expression by *M. crystallinum* between high nutrient and low nutrient conditions (Figure 5). Paul and Cockburn (1990) applying adequate N and P supply to young seedlings, leading to sufficient photosynthesis and carbohydrate accumulation, found a positive response of CAM expression in adults of *M. crystallinum* to N and P deficiency. In our study, low nutrient availability (one‐third of high nutrient conditions) was applied during all stages; in low water conditions, photosynthesis and production of carbohydrate are mainly limited by soil moisture instead of nutrients (Figures 3 and 4), implying that low nutrient supply is still adequate relative to water conditions and could sustain sufficient carbohydrate for nocturnal carboxylation. Although low nutrient supply was a limiting factor of plant photosynthesis and productivity in high water conditions (Figures 3 and 4), CAM expression in *M. crystallinum* did not occur because of lack of water stress (Figure 5; Osmond, 1978; Winter & Holtum, 2007, 2014). These results were in line with the studies that showed that CAM was best expressed in facultative *Kalanchoe lateritia* at moderately low N conditions (i.e., with N applications 20% of the reference rate) as compared to ambient and very low N availability, likely because sufficient carbohydrate and environmental stress (i.e., N deficiency) were both satisfied under moderately low N (Santos & Salema 1991, 1992).

This study investigated the ecophysiological mechanisms underlying the potential advantage of a model facultative CAM species (*M. crystallinum*) while interacting with a C_3_ species (*B. mollis*) found in California's coastal grasslands under nutrient and water manipulations. We found that because of its drought tolerance, *M. crystallinum* had a competitive advantage relative to *B. mollis* in low water and N deposition conditions. In high water conditions, however, *B. mollis* was a stronger competitor for soil nutrients and light resources. This strong competition restricted the ability of *M. crystallinum* to switch to CAM expression—a type of physiological plasticity used by *M. crystallinum* to adapt to environmental stress and to increase seed production and plant fitness. With an increasing trend of drought and nitrogen deposition in the coming decades, we suggest that the dominance of CAM plants is likely to increase in drylands in a future environment. The lack of ability to adapt to strong competition by switching to CAM photosynthesis in high water conditions suggests that the potential of facultative CAM plants such as *M. crystallinum* to expand to wet environments would be restricted.

## AUTHOR CONTRIBUTIONS

K.Y., P.D., and S.C. designed the research; K.Y. developed and performed experiments; K.Y., D.C., and A.P. analyzed data; K.Y. wrote the manuscript; and all others contributed to revisions.
