# Supplementary material for: The effect of nitrogen availability and water conditions on competition between a facultative CAM plant and an invasive grass
Source: Ecol Evol. 2017 Aug 23;7(19):7739–49. doi: 10.1002/ece3.3296 (PMC5632618; doi:10.1002/ece3.3296)
Supplement: Supplementary file 1 [file ECE3-7-7739-s001.docx]

**Supporting information 1: Aboveground plant nitrogen (APN)**

This supporting information provides aboveground plant nitrogen (APN) as affected by nutrient, water, species, and time as well as their interactions. Increase in nutrient availability significantly increased APN for all the vegetation types (FC, FMC, G, MG) regardless of water conditions (all P ≤ 0.0082, Fig. S1) except for the cases of FC and FMC in low water conditions in the second and third stages (all P ≥ 0.0863, Fig. S1a, b). APN for CAM in mixture (FCM) was significantly lower than that in CAM alone (FC) regardless of nutrient and water availability (all P < 0.0001, Fig. S1), except for low water conditions in the second and third stages (all P ≥ 0.1602, Fig. S1b, c) in which grasses died and thus competition did not occur.


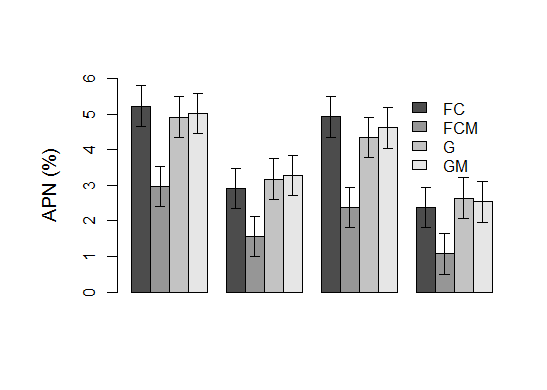


a


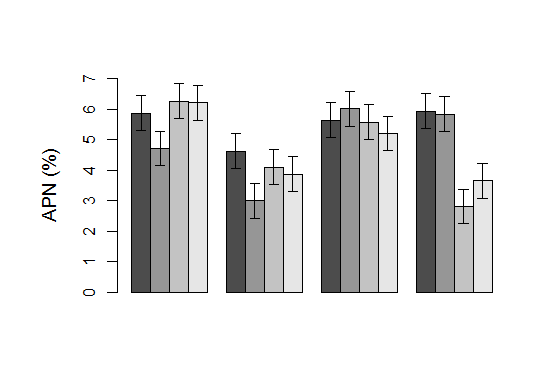

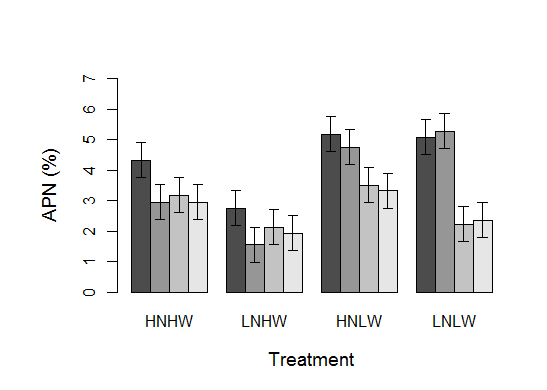


b

c

Fig. S1. Aboveground plant nitrogen (APN) in *Mesembryanthemum crystallinum* alone (FC), *M. crystallinum* in mixture (FCM), *Bromus mollis* alone (G), *B. mollis* in mixture (GM) under different nutrient and water conditions in the first (a), second (b), and third (c) stages of the experiment. HNHW refers to high nutrient and high water conditions; LNHW refers to low nutrient and high water conditions; HNLW refers to high nutrient and low water conditions; LNLW refers to low nutrient and low water conditions. Each bar represents the mean of 6 values while error bars indicate 95% confidence intervals.

**Supporting information 2: Relative Light Intensity (RLI)**

This supporting information provides the results of relative light intensity (RLI) for CAM in mixture as affected by nutrient and water conditions. In high water conditions, light availability for *M. crystallinum* in mixture was significantly lower than for *M. crystallinum* alone, as shown by relative light intensity (RLI) being much less than 100 % (Fig. S2). The increase in nutrient availability in high water conditions increased the biomass of *B. mollis,* and shade effects, thus leading to a decrease in RLI. In low water conditions, *B. mollis* died in the second and third stages, and thus did not have shade effects on *M. crystallinum* in mixture (Fig. S2).


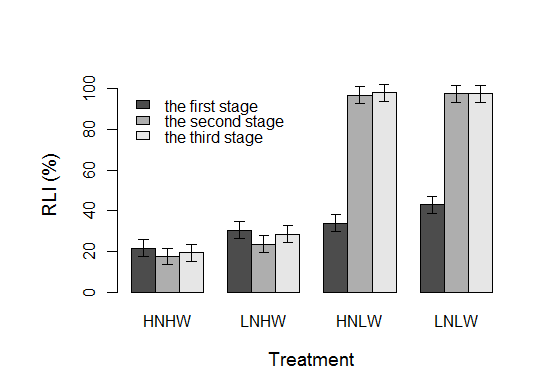


Fig. S2. Relative light intensity (RLI) for *Mesembryanthemum crystallinum* in mixture as affected by nutrient and water conditions in the first, second, and third stages. Symbols for each treatment are the same as Fig. S1. Each bar represents the mean of 6 values while error bars indicate 95% confidence intervals.

**Supporting information 3: Specific Leaf Area (SLA)**

This supporting information provides the results of specific leaf area (SLA) as affected by nutrient, water, species, and time as well as their interactions. The significant interactive effect of N × water shows that the effect of nutrient on specific leaf area (SLA) depends on water conditions (Table 1; P<0.001 for N × water). In fact, in high water conditions SLA of all vegetation types (FC, FMC, G, MG) in the high nutrient treatment was significantly greater than in the low nutrient treatment (all P ≤ 0.0430, Fig. S3) but this was not the case for grass in mixture in the second stage (P = 0.2385, Fig. S3b) and CAM in mixture in the third stage (P = 0.2655, Fig. S3c). In contrast, in low water conditions SLA of all vegetation types (FC, FMC, G, MG) in the high nutrient treatment was not significantly different from that in the low nutrient treatment (all P ≥ 0.2946, Fig. S3) except for CAM plants mixed with grasses in the third stage (P = 0.0367, Fig. S3c). SLA in high water conditions was generally higher than low water conditions (all P ≤ 0.0111, Fig. S3). In low water conditions SLA of CAM alone was significantly lower than CAM in mixture in the second and third stages (P = 0.0006 for the second stage; P < 0.0001 for the third stage, Fig. S3b, c). In high water conditions SLA of CAM alone was generally the same with CAM in mixture (all P ≥ 0.1165, Fig. S3).

a


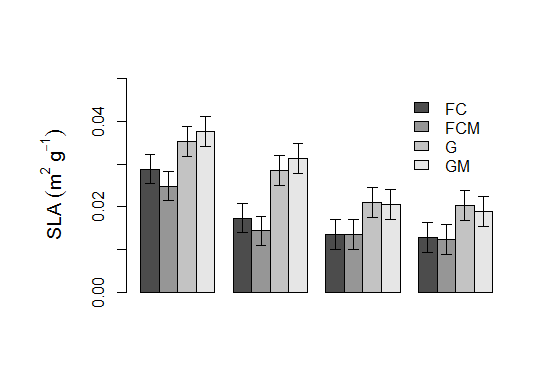

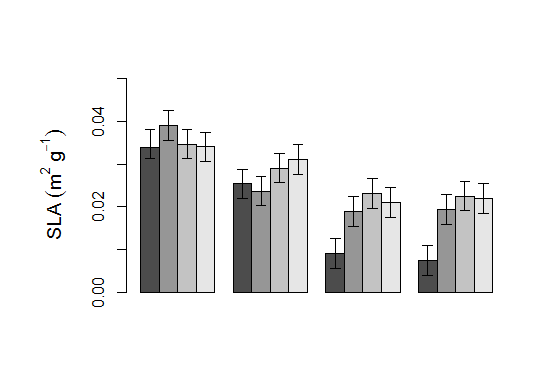

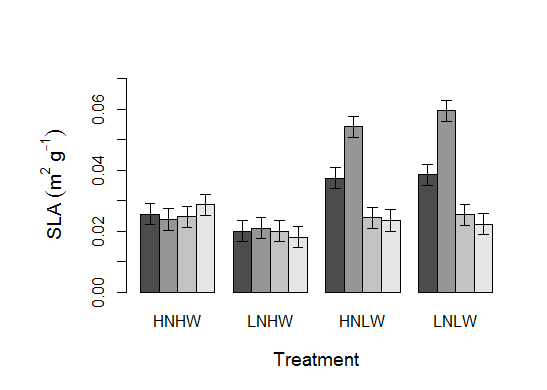


c

b

Fig. S3. Specific leaf area (SLA) in *Mesembryanthemum crystallinum* alone (FC), *M. crystallinum* in mixture (FCM), *Bromus mollis* alone (G), *B. mollis* in mixture (GM) under different nutrient and water conditions in the first (a), second (b), and third (c) stages of the experiment. Symbols for each treatment are the same as Fig. S1. Each bar represents the mean of 6 values while error bars indicate 95% confidence intervals.

**Supporting information 4: Belowground/aboveground biomass ratio (BA)**

Increased nutrient availability significantly reduced belowground/aboveground biomass ratio (BA) of *M. crystallinum* alone (FC), *B. mollis* alone (G) and *B. mollis* in mixture (GM) in both low water and high water conditions in all the three stages (all P < 0.0001, Fig. S4). Similarly, BA of FC, G, and GM in high water conditions was generally lower than that in low water conditions. Overall, these results supported the root functional equilibrium hypothesis that plants growing in conditions of higher nutrient and/or water availability invest more in aboveground biomass and less in roots. *M. crystallinum* alone and *M. crystallinum* in mixture featured a low BA and had a significantly lower BA than *B. mollis* alone and in mixture in all the treatments over the three stages (all P < 0.0001, Fig. S4).


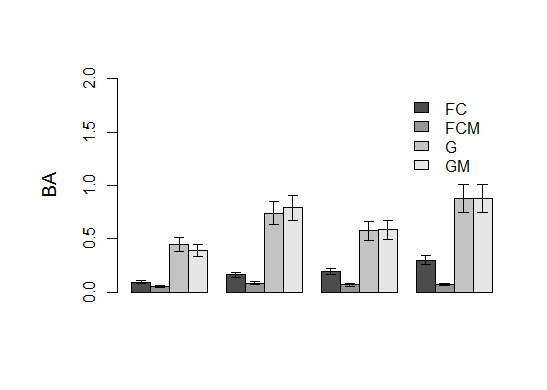

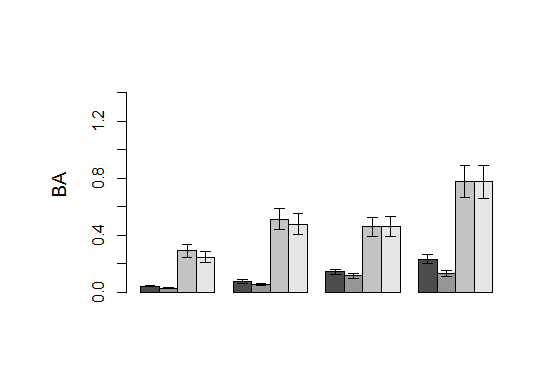

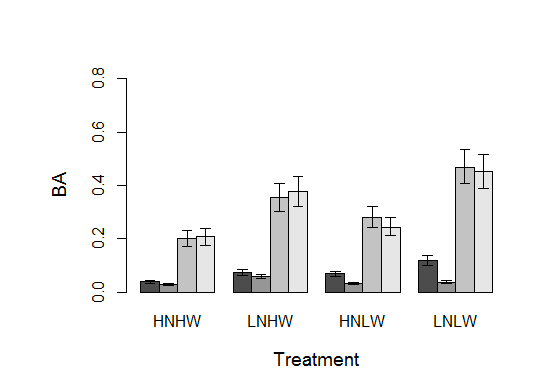


a

b

c

Fig. S4. Belowground/aboveground biomass ratio (BA) in *Mesembryanthemum crystallinum* alone (FC), *M. crystallinum* in mixture (FCM), *Bromus mollis* alone (G), *B. mollis* in mixture (GM) under different nutrient and water conditions in the first (a), second (b), and third (c) stages of the experiment. Symbols for each treatment are the same as in Fig. S1. The BA values were natural log transformed prior to ANOVA. Each bar represents the mean of 6 values while the error bars indicate 95% confidence intervals.
